# Effect of Oral Administration Involving a Probiotic Strain of *Lactobacillus reuteri* on Pro-Inflammatory Cytokine Response in Patients with Chronic Periodontitis

**DOI:** 10.1007/s00005-014-0277-y

**Published:** 2014-02-09

**Authors:** Anna K. Szkaradkiewicz, Janina Stopa, Tomasz M. Karpiński

**Affiliations:** 1 Department of Conservative Dentistry and Periodontology, University of Medical Sciences, Poznań, Poland; 2 Department of Medical Microbiology, University of Medical Sciences, Wieniawskiego 3, 61-712 Poznań, Poland

**Keywords:** Chronic periodontitis, Lactobacillus, Cytokines, Inflammation, Immunomodulatory effect

## Abstract

This study aimed at evaluation of pro-inflammatory cytokine response (TNF-α, IL-1β and IL-17) in patients with chronic periodontitis administered *per os* with a probiotic strain of *Lactobacillus reuteri*. In the 38 adult patients with moderate chronic periodontitis, professional cleaning of teeth was performed. Two weeks after performing the oral hygienization procedures, clinical examination permitted to distinguish a group of 24 patients (Group 1) in whom treatment with probiotic tablets containing *L. reuteri* strain, producing hydrogen peroxide (Prodentis), was conducted. In the remaining 14 patients, no probiotic tablet treatment was applied (the control group; Group 2). From all patients in two terms, gingival crevicular fluid (GCF) was sampled from all periodontal pockets. Estimation of TNF-α, IL-lβ and IL-17 in GCF was performed using the ELISA method. After completion of the therapy with probiotic tablets, 18 (75 %) of the patients of Group 1 have manifested a significant decrease in levels of studied pro-inflammatory cytokines (TNF-α, IL-1β and IL-17). In parallel, we have detected an improvement of clinical indices [sulcus bleeding index (SBI), periodontal probing depth (PPD), clinical attachment level (CAL)]. At individuals of Group 2 levels of studies, pro-inflammatory cytokines and clinical indices (SBI, PPD, CAL) were significantly higher than in Group 1. Results obtained in this study indicate that application of oral treatment with tablets containing probiotic strain of *L. reuteri* induces in most patients with chronic periodontitis a significant reduction of pro-inflammatory cytokine response and improvement of clinical parameters (SBI, PPD, CAL). Therefore, such an effect may result in a reduced activity of the morbid process.

## Introduction

Chronic periodontitis represents a destructive inflammatory disease, encompassing gingiva, radical cementum, periodontitis and osseous alveolar process. The disease develops most frequently in adults and is characterized by a moderate or severe clinical course. Epidemiological studies indicate that chronic periodontitis belongs to the most frequent chronic diseases in humans. In Western Europe, around 36 % persons aging 35–44 years manifest moderate and around 10 % severe form of chronic periodontitis. In countries of Eastern Europe, the fractions are higher and amount to 45 and 30–40 %, respectively (Sheiham and Netuveli 2002).

Microbiological and experimental studies on an animal model indicate that, in etiopathogenesis of periodontitis, principal role is played by pathogenic anaerobic bacteria, defined as periodontopathogens (Kebschull and Papapanou 2011). The prevailing periodontopathogen involves *Porphyromonas gingivalis* (Kim et al. 2010; Socransky and Haffajee 2005). Moreover, the species of *Tannerella forsythia*, *Treponema denticola*, *Fusobacterium nucleatum*, *Prevotella intermedia*, and also the relative anaerobe of *Aggregatibacter actinomycetemcomitans*, belong to significant etiopathogenic factors of the periodontal disease in adults (Colombo et al. 2006; Sbordone et al. 2000). However, periodontopathogens may also represent a physiological component of oral cavity microflora in healthy individuals (Ledder et al. 2007). At the same time, in recent investigations, oral lactobacilli have been demonstrated to inhabit periodontal pockets in periodontitis patients with a significantly higher frequency in moderate, as compared to severe form of the disease (Szkaradkiewicz et al. 2006; Szkaradkiewicz and Stopa 2008). Moreover, the data available till now indicate that bacilli of *Lactobacillus* spp. may modify composition of oral cavity microflora through antagonistic interactions against potentially pathogenic species and, in particular, they may inhibit growth of certain periodontopathogens (Teanpaisan et al. 2011; Testa et al. 2003; Servin 2004; Van Hoogmoed et al. 2008). Therefore, the bacteria may protect tissue of periodontium restricting growth of pathogenic bacteria. On the other hand, it has already been well documented that pathogenesis of chronic periodontitis may be determined by a pro-inflammatory response of cytokines, expressed in particular by secretion of tumor necrosis factor (TNF)-α, interleukin (IL)-1β and IL-17. The cytokines play a particular role in induction and development of local inflammatory response. TNF-α and IL-1β, produced mainly by monocytes/macrophages promote a inflammatory host reaction, mobilizing several processes, such as an increased expression of adhesion molecules on vascular endothelial cells, stimulated chemokinase production by connective tissue and endothelial cells and also release of other mediators (Bradley 2008; Dinarello 2009; Hanada and Yoshimura 2002). In turn, IL-17, produced by activated lymphocytes Th17, provides neutrophiles recruitment to the inflammatory focus and, in parallel, cooperates with TNF-α and IL-1β (Gaffen 2011). Studies of recent years have demonstrated that high levels of TNF-α, IL-1β and IL-17 in periodontal pockets, may determine clinical form of the disease (Santos et al. 2010; Szkaradkiewicz et al. 2011).

Considering the above, this study aimed at evaluation of pro-inflammatory cytokine response (TNF-α, IL-1β and IL-17) in patients with chronic periodontitis administered *per os* with a probiotic strain of *Lactobacillus reuteri*.

## Materials and Methods

The studies were conducted on 38 adults (20 women and 18 men) recruited from among patients of the Department of Conservative Dentistry and Periodontology, University of Medical Sciences in Poznań. The patients qualified for the studies were clinically healthy and in anamnesis they reported no systemic diseases, administered within recent 3 weeks no anti-bacterial drugs and non-smokers. Within the year preceding the studies, the patients were not subjected to periodontological treatment, nor did they use additional means of oral hygiene, such as dental thread, anti-septic liquids, and irrigators. The investigated group included individuals with a moderate chronic periodontitis, 31–46 years of age. Mean duration of the disease amounted to 19.4 ± 2.8 months (18–27 months).

The clinical examination was performed by a single specialized dentist; all data of anamnesis and physical examination were documented in individual files of the patients. In the patients, professional cleaning of teeth was performed accompanied by scaling and root planing and instruction related to maintenance of oral cavity hygiene. Scaling and root planing was done in the whole oral cavity by means of manual and ultrasound tools, avoiding the use of antiseptics. Two weeks after performing the oral hygienization procedures, clinical examination permitted to distinguish a group of 24 patients (Group 1) in whom treatment with probiotic bacteria was conducted. The remaining 14 patients with a significant decrease in clinical indices following the hygienization procedure were not subjected to treatment with the probiotic tablets (Group 2). The criteria excluding patients from the treatment with probiotic tablets were accepted to include a statistically significant decrease in clinical indices [sulcus bleeding index (SBI), periodontal probing depth (PPD), clinical attachment level (CAL)] following the hygienization procedure. From all patients of Group 1, material for laboratory studies i.e., gingival crevicular fluid (GCF) was sampled from all periodontal pockets (first time point of the studies). The diet supplement in the form of probiotic bacteria suction tablets, containing *L. reuteri* strain producing hydrogen peroxide (10^8^ CFU *L. reuteri* ATCC PTA 5289, Prodentis) was administered to the patients twice daily, after tooth brushing. Two weeks after termination of probiotic tablet administration, the material for testing was sampled again (second time point of the studies).

The research project received consent of the Bioethical Commission by the University of Medical Sciences in Poznań.

### Clinical Diagnosis

The clinical diagnosis encompassed anamnesis and dental physical examination. The anamnesis pertained to manifestation of systemic diseases and use of anti-bacterial drugs within the recent 3 weeks and cigarette smoking by the patients. In every patient, oral cavity hygiene, condition of gingivae, depth of periodontal pockets and loss of epithelial attachment were evaluated.

Oral cavity hygiene was evaluated using plaque index (Pl.I) according to Silness and Löe (1964). Condition of gingivae was appraised using gingival index (GI) (Löe 1967; Silness and Löe 1964) and the SBI (Műhlemann and Son 1971). Loss of periodontal tissue due to periodontal disease was quantitated measuring PPD and CAL. PPD and CAL were measured on six surfaces by all the remaining in oral cavity teeth using a scaled periodontological WHO 621 Hu-Friedy probe (the scale ranged up to 11.5).

Results of physical examination provided grounds for diagnosis of a periodontal disease. Criteria of a simplified classification of gingival and periodontal disease were accepted, which took into account degree of advancement manifested by chronic periodontitis. Moderate chronic periodontitis was diagnosed when: GI >0, SBI >0, CAL >5 mm and PPD >4 mm, in cases of two or more non-neighboring teeth (Armitage 1999; Górska 2007; World Health Organization 1997).

### Sampling of GCF

The investigated material involved samples of gingival crevicular fluid. The GCF was sampled using Hamilton 25 μl syringes (Hamilton, USA) with a thin, endodontal needle (0.3 mm in diameter, 25 mm in length) from all periodontal pockets following blocking access of saliva. Before sampling of the GCF, the patients were not subjected to hygienization procedures. The needle was introduced to periodontal pockets, carefully sampling the GCF using suction. GCF was sampled from the most pathologically active crevicular pockets (4–6 pockets). If needed, during sampling of GCF, 1–2 min intervals were used to allow for GCF inflow into the pocket. From every patient, 60 μl GCF was sampled in the time required to obtain the appropriate volume of the fluid. Subsequently, the GCF was placed in sterile Eppendorf tubes, labeled with numbers corresponding to patient’s file number (Champagne et al. 2003; Griffiths 2003). The obtained GCF was divided into three batches assigned for immunological studies. Till the time of testing of their cytokine content they were stored in the temperature of −70 °C.

### Estimation of Cytokines

#### Estimation of TNF-α in GCF

Estimation of TNF-α in GCF (at 1:10 dilution in PBS) was performed using the immunoenzymatic (ELISA) technique, taking advantage of high sensitivity Quantikine HS ELISA Human TNF-α (R&D Systems, USA) kits, manifesting mean minimum detectability (MDD) of 0.106 pg/ml. Value of absorbance at the wavelength of *A* = 490 nm was obtained using Reader 250 (bioMerieux, France). The final result of the studied cytokine concentration involved a product of the readout on the standard curve and the applied dilution (×10).

#### Estimation of IL-1β in GCF

Estimation of IL-lβ in GCF (at 1:10 dilution in PBS) was performed using immunoenzymatic technique (ELISA) taking advantage of high sensitivity kits of Quantikine HS ELISA Human IL-lβ/IL-1F2 (R&D Systems, USA), manifesting MDD of 0.057 pg/ml. Value of absorbance was recorded at the wavelength of *A* = 490 nm using Reader 250 (bioMerieux, France). The final result of the studied cytokine concentration involved a product of the readout on the standard curve and the applied dilution (×10).

#### Estimation of IL-17 in GCF

Estimation of IL-6 in GCF (at 1:10 dilution in PBS) using immunoenzymatic technique (ELISA) took advantage of Human IL-17 Platinum ELISA kits (eBioscience, USA), manifesting MDD of 0.5 pg/ml. Value of absorbance was recorded at the wavelength of *A* = 450 nm using Reader 250 (bioMerieux, France). The results were read out following preparation of a standard curve. The final result of the studied cytokine concentration involved a product of the readout on the standard curve and the applied dilution (×10).

### Statistical Methods

Results obtained in the studies were subjected to statistical analysis employing the computer Statistica 8 software for the Windows operational system. Analysis of clinical indices (Pl.I, GI, SBI, PPD, CAL) took advantage of the nonparametric Mann–Whitney’s test and the test of Kruskal–Wallis. In comparative analysis of cytokines levels in the studied groups, the nonparametric test of Kruskal–Wallis with the test of Dunn was employed. The difference was considered to be significant at *p* < 0.05.

## Results

### Results of Dental Examination

At the first time point of the studies, before treatment with probiotic tablets in the first group of patients, the values of clinical indices (Pl.I, GI, SBI, PPD, CAL) were obtained, which are presented in Table 1. Values of clinical parameters obtained at the second time point of the studies (after completion of treatment using probiotic tablets), as compared to values obtained at the first time point, in 18 patients manifested a significant decrease in mean values of SBI, PPD and CAL (subgroup 1A). The most pronounced difference, involving over 20 % reduction of the index value, was detected in examination of SBI. In the remaining six patients, values of the indices manifested no significant differences between the two time points of the studies (subgroup 1B).

**Table 1:** Values of clinical indices (mean ± SD) in patients with moderate chronic periodontitis in first and second term of study

Clinical indices	First term of study		Second term of study		
	Group 1 (*n* = 24)	Group 2 (*n* = 14)	Group 1 (*n* = 24)		Group 2 (*n* = 14)
			Subgroup 1a (*n* = 18)	Subgroup 1b (*n* = 6)	
PL.I	1.61 ± 0.31	1.64 ± 0.29	1.65 ± 0.26	1.76 ± 0.38	1.72 ± 0.34
GI	1.33 ± 0.29	1.36 ± 0.31	1.21 ± 0.36	1.29 ± 0.24	1.31 ± 0.27
SBI	1.69 ± 0.35	1.73 ± 0.32	1.24 ± 0.31*	1.67 ± 0.36	1.75 ± 0.31
PPD	3.35 ± 0.32	3.39 ± 0.36	3.06 ± 0.35*	3.26 ± 0.45	3.34 ± 0.38
CAL	3.47 ± 0.38	3.49 ± 0.35	3.16 ± 0.27*	3.53 ± 0.34	3.56 ± 0.41

* The difference between the first and the second term is statistically significant in a given group of patients

### Results of Testing Levels Manifested by Cytokines

At the first time point of the studies in 24 patients (Group 1), cytokine levels in GCF were as follows: TNF-α: 5.52 ± 0.94; IL-1β: 20.74 ± 2.71 and IL-17: 17.58 ± 3.23. In the second time point in all participants of subgroup 1A, a significant decrease in studied cytokine levels was detected, as compared to levels noted at the first time point. In turn, all individuals of subgroup 1B manifested no significant decrease in mean levels of studied cytokines. In the subgroup 1A, patients treated with tablets containing *L. reuteri* strain manifested an around double decrease in levels of TNF-α and IL-17 and an around triple decrease in the level of IL-1β. The results obtained for TNF-α, IL-1β and IL-17 in GCF of Group 1 and Group 2 patients at the two time points of the studies are presented in Table 2.

**Table 2:** Levels of TNF-α, IL-1β and IL-17 (pg/ml) in GCF of patients in first and second term of study

Clinical indices	First term of study		Second term of study		
	Group 1 (*n* = 24)	Group 2 (*n* = 14)	Group 1 (*n* = 24)		Group 2 (*n* = 14)
			Subgroup 1A (*n* = 18)	Subgroup 1B (*n* = 6)	
TNF-α (pg/ml)	5.52 ± 0.94	5.42 ± 0.87	2.34 ± 0.87*	5.49 ± 0.84	5.27 ± 0.94
IL-1β (pg/ml)	20.74 ± 2.71	20.16 ± 2.46	6.83 ± 1.51*	19.86 ± 1.98	19.63 ± 2.21
IL-17 (pg/ml)	17.58 ± 3.23	17.23 ± 3.15	9.35 ± 1.71*	16.62 ± 2.29	15.93 ± 2.37

* The difference between the first and the second term is statistically significant in a given group of patients

## Discussion

In the studies, an attempt was made to evaluate therapeutic application in patients with chronic periodontitis of oral probiotic tablets containing the hydrogen peroxide-producing strain of *L. reuteri*. This strain represents one of the already well-recognized probiotic bacteria species with a documented action in several bacterial infections (Hunter et al. 2012; Liu et al. 2010; Prince et al. 2012; Szajewska et al. 2013). Reuterin, produced by the strain, represents 3-hydroxypropionaldehyde of a broad spectrum of an anti-bacterial activity (Cadieux et al. 2008). It acts in a broad pH range and is resistant to action of lipo- and proteolytic enzymes. It also suppresses production of pro-inflammatory cytokines. Reuterin blocks adherence and prevents against pathogen colonization (Bian et al. 2011; Jones and Versalovic 2009; Kang et al. 2011). Oral tablets containing the probiotic strain of *L. reuteri* were used earlier in patients with chronic periodontitis by Vivekananda et al. (2010). The authors demonstrated that oral administration of *L. reuteri* strain twice daily in 30 adult patients with chronic periodontitis was followed by a significant reduction in clinical indices, including Pl.I, GI and SBI. Moreover, the study demonstrated that treatment with the probiotic strain of *L. reuteri* significantly reduced numerical force of analyzed periopathogens in subgingival plaque (Vivekananda et al. 2010). A reduction in number of selected periodontal pathogens in subgingival plaque was detected also in patients with gingivitis (Iniesta et al. 2012).

In our studies, oral probiotic tablets have been applied, containing the hydrogen peroxide-producing strain of *L. reuteri*, in 24 adults with moderate periodontitis (Group 1). After completion of the therapy, 18 patients (subgroup 1A) have manifested a significant clinical improvement manifested in decreased values of clinical indices, including SBI, PPD, CAL, which corresponds to the above presented data. In turn, in six patients (subgroup 1B) no significant improvement in clinical indices has been detected. This may indicate that no effective colonization occurred by the applied probiotic strain of *L. reuteri*. It remains also possible that in some patients the process of periodontium colonization requires longer administration of probiotic tablets containing *L. reuteri* strain.

In parallel, in our study, we have evaluated for the first time the cytokine pro-inflammatory response in patients with the moderate form of chronic periodontitis, in whom treatment with *L. reuteri* probiotic strain was implemented. In the consequence of the applied therapy, a significant reduction in levels of TNF-α, IL-1β and IL-17 has been noted in 18 patients (75 %). On the other hand, in the remaining six patients (25 %) forming the subgroup 2B, no clinical improvement has been detected and levels of estimated pro-inflammatory cytokines (TNF-α, IL-1β and IL-17) have not changed significantly. Moreover, levels of studied cytokines in patients of Group 2, who did not obtain probiotic tablets, were significantly higher as compared to patients of subgroup 1A, while they did not differ from the levels detected in the subgroup 1B.

TNF-α exhibits pro-inflammatory properties, it affects also osteoclastogenesis, maturation of osteoclasts and bone resorption. Initiating the inflammatory process it mobilizes also the mechanism of destruction in periodontal tissues (Bradley 2008). Concentration of TNF-α in GCF was found to be significantly higher in persons with periodontitis than in individuals with healthy periodontium (Kurtiş et al. 2005). IL-1β plays also a significant role in development of inflammatory process, including that in periodontal tissues (Hou et al. 2003). In patients with chronic periodontitis, concentration of the cytokine in GCF is significantly higher than in patients with healthy periodontium (Yücel et al. 2008). IL-17 exerts a regulatory influence on a local inflammatory response. IL-17 makes possible neutrophiles recruitment to the inflammatory focus and cooperates with other pro-inflammatory cytokines, mainly with TNF-α and IL-1β (Gaffen 2011).

As shown by data of recent years, the pro-inflammatory cytokine response may play a significant role in nonspecific response against bacterial and fungal pathogens constituting also a principal mediator of periodontal disease (Kowalski et al. 2006; Okada and Murakami 1998). Moreover, intensity of the pro-inflammatory cytokine response seems to determine the severe and moderate clinical form of chronic periodontitis (Passoja et al. 2010; Santos et al. 2010; Szkaradkiewicz et al. 2011). The detected in this study, following treatment with the probiotic strain of *L. reuteri*, decreased levels of TNF-α, IL-1β and IL-17 in periodontal pockets of patients with periodontitis may carry a clinical significance, preventing against progression of the disease. The obtained results allow also to conclude that the probiotic strain of *L. reuteri* induces a decrease in the pro-inflammatory cytokine response in chronic periodontitis. The conclusion is supported by experimental studies indicating that probiotic strains of *Lactobacillus* may exert a potential immunomodulating effect, suppressing expression of genes coding for pro-inflammatory cytokines (Servin 2004; Vissers et al. 2011). In the presented investigations, we have demonstrated for the first time the beneficial significance of oral tablets, containing *L. reuteri* in treatment of chronic periodontitis. The obtained results prompt us to undertake further clinical studies with use of placebo.

In conclusion, results obtained in the study indicate that application of oral treatment with tablets containing the probiotic strain of *L. reuteri* induces in most of the patients with chronic periodontitis a significant reduction in pro-inflammatory cytokine response and improvement of clinical parameters (SBI, PPD, CAL). Therefore, such an action may reduce the activity of the morbid process.

## References

[CR1] Armitage GC (1999). Development of a classification system for periodontal diseases and conditions. Ann Periodontol.

[CR2] Bian L, Molan AL, Maddox I (2011). Antimicrobial activity of *Lactobacillus reuteri* DPC16 supernatants against selected food borne pathogens. World J Microbiol Biotechnol.

[CR3] Bradley JR (2008). TNF-mediated inflammatory disease. J Pathol.

[CR4] Cadieux P, Wind A, Sommer P (2008). Evaluation of reuterin production in urogenital probiotic *Lactobacillus reuteri* RC-14. Appl Environ Microbiol.

[CR5] Champagne CM, Buchanan W, Reddy MS (2003). Potential for gingival crevice fluid measures as predictors of risk for periodontal diseases. Periodontol 2000.

[CR6] Colombo AV, Silva CM, Haffajee A (2006). Identification of oral bacteria associated with crevicular epithelial cells from chronic periodontitis lesions. J Med Microbiol.

[CR7] Dinarello CA (2009). Immunological and inflammatory functions of the interleukin-1 family. Annu Rev Immunol.

[CR8] Gaffen SL (2011). Recent advances in the IL-17 cytokine family. Curr Opin Immunol.

[CR9] Górska R (2007). Guideline for diagnosis of periodontal diseases (in Polish). Dent Med Probl.

[CR10] Griffiths GS (2003). Formation, collection and significance of gingival crevice fluid. Periodontol.

[CR11] Hanada T, Yoshimura A (2002). Regulation of cytokine signaling and inflammation. Cytokine Growth Factor Rev.

[CR12] Hou LT, Liu CM, Liu BY (2003). Interleukin-1β, clinical parameters and matched cellular-histopathologic changes of biopsied gingival tissue from periodontitis patients. J Periodontal Res.

[CR13] Hunter C, Dimaguila MA, Gal P (2012). Effect of routine probiotic, *Lactobacillus reuteri* DSM 17938, use on rates of necrotizing enterocolitis in neonates with birthweight <1000 grams: a sequential analysis. BMC Pediatr.

[CR14] Iniesta M, Herrera D, Montero E (2012). Probiotic effects of orally administered *Lactobacillus reuteri*-containing tablets on the subgingival and salivary microbiota in patients with gingivitis. A randomized trial. J Clin Periodontol.

[CR15] Jones SE, Versalovic J (2009). Probiotic *Lactobacillus reuteri* biofilms produce antimicrobial and anti-inflammatory factors. BMC Microbiol.

[CR16] Kang MS, Oh JS, Lee HC (2011). Inhibitory effect of *Lactobacillus reuteri* on periodontopathic and cariogenic bacteria. J Microbiol.

[CR17] Kebschull M, Papapanou PN (2011). Periodontal microbial complexes associated with specific cell and tissue responses. J Clin Periodontol.

[CR18] Kim YC, Ko Y, Hong SD (2010). Presence of *Porphyromonas gingivalis* and plasma cell dominance in gingival tissues with periodontitis. Oral Dis.

[CR19] Kowalski J, Górska R, Dragan M (2006). Clinical state of the patients with periodontitis, IL-1 polymorphism and pathogens in periodontal pocket—is there any link? (An introductory report). Adv Med Sci.

[CR20] Kurtiş B, Tüter G, Serdar M (2005). Gingival crevicular fluid levels of monocyte chemoattractant protein-1 and tumor necrosis factor-α in patients with chronic and aggressive periodontitis. J Periodontol.

[CR21] Ledder RG, Gilbert P, Huws SA (2007). Molecular analysis of the subgingival microbiota in health and disease. Appl Environ Microbiol.

[CR22] Liu Y, Fatheree NY, Mangalat N (2010). Human-derived probiotic *Lactobacillus reuteri* strains differentially reduce intestinal inflammation. Am J Physiol Gastrointest Liver Physiol.

[CR23] Löe H (1967). The gingival index, the plaque index and the retention index system. J Periodontol.

[CR24] Műhlemann HR, Son S (1971). Gingival sulcus bleeding a leading symptom in initial gingivitis. Helv Odont Acta.

[CR25] Okada H, Murakami S (1998). Cytokine expression in periodontal health and disease. Crit Rev Oral Biol Med.

[CR26] Passoja A, Puijola I, Knuuttila M (2010). Serum levels of interleukin-10 and tumour necrosis factor-α in chronic periodontitis. J Clin Periodontol.

[CR27] Prince T, McBain AJ, O’Neill CA (2012). *Lactobacillus reuteri* protects epidermal keratinocytes from *Staphylococcus aureus*-induced cell death by competitive exclusion. Appl Environ Microbiol.

[CR28] Santos VR, Ribeiro FV, Lima JA (2010). Cytokine levels in sites of chronic periodontitis of poorly controlled and well-controlled type 2 diabetic subjects. J Clin Periodontol.

[CR29] Sbordone L, Di Genio M, Bortolaia C (2000). Bacterial virulence in the etiology of periodontal diseases. Minerva Stomatol.

[CR30] Servin AL (2004). Antagonistic activities of lactobacilli and bifidobacteria against microbial pathogens. FEMS Microbiol Rev.

[CR31] Sheiham A, Netuveli GS (2002). Periodontal diseases in Europe.

[CR32] Silness J, Löe H (1964). Periodontal disease in pregnancy. II. Correlation between oral hygiene and periodontal condition. Acta Odontol Scand.

[CR33] Socransky SS, Haffajee AD (2005). Periodontal microbial ecology.

[CR34] Szajewska H, Gyrczuk E, Horvath A (2013). *Lactobacillus reuteri* DSM 17938 for the management of infantile colic in breastfed infants: a randomized, double-blind, placebo-controlled trial. J Pediatr.

[CR35] Szkaradkiewicz AK, Stopa J (2008). *Lactobacillus* spp. of oral cavity microflora in chronic periodontitis. Pol J Environ Stud.

[CR36] Szkaradkiewicz AK, Tukiendorf B, Stopa J (2006). Oral lactobacilli and periodontitis. Clin Microbiol Infect.

[CR37] Szkaradkiewicz AK, Karpiński TM, Zeidler A (2011). Protective effect of oral lactobacilli in pathogenesis of chronic periodontitis. J Physiol Pharmacol.

[CR38] Teanpaisan R, Piwat S, Dahlèn G (2011). Inhibitory effect of oral *Lactobacillus* against oral pathogens. Lett Appl Microbiol.

[CR39] Testa MM, de Valladares R, de Cardenas IL (2003). Antagonistic interactions among *Fusobacterium nucleatum* and *Prevotella intermedia* with oral lactobacilli. Res Microbiol.

[CR40] Van Hoogmoed CG, Geertsema-Doombusch GI, Teughels W (2008). Reduction of periodontal pathogens adhesion by antagonistic strains. Oral Microbiol Immunol.

[CR41] Vissers YM, Snel J, Zuurendonk PF (2011). *Lactobacillus* strains differentially modulate cytokine production by *Lactobacillus* hPBMC from pollen-allergic patients. FEMS Immunol Med Microbiol.

[CR42] Vivekananda MR, Vandana KL, Bhat KG (2010). Effect of the probiotic Lactobacilli reuteri (Prodentis) in the management of periodontal disease: a preliminary randomized clinical trial. J Oral Microbiol.

[CR43] World Health Organization (1997). Oral Health Surveys: basic methods.

[CR44] Yücel OO, Berker E, Gariboğlu S (2008). Interleukin-11, interleukin-1β, interleukin-12 and the pathogenesis of inflammatory periodontal diseases. J Clin Periodontol.

